# Enhancing Medical Student Experience in Psychiatry Placement in Stockport: A Quality Improvement Project

**DOI:** 10.1192/bjo.2024.358

**Published:** 2024-08-01

**Authors:** Nafisa Darod

**Affiliations:** Greater Manchester Mental Health Trust, Manchester, United Kingdom

## Abstract

**Aims:**

Fourth-year medical students from Manchester University undergo a four-week Psychiatry rotation in Stockport as part of their curriculum. Placed in both community and inpatient teams within General Adult and Older Adult Psychiatry services, this placement offers a unique opportunity for students to gain clinical and educational experience in Psychiatry, potentially shaping their perception of the field. This quality improvement project aimed to enhance the overall experience of medical students during their Psychiatry placement in Stockport.

**Methods:**

A retrospective review of quantitative and qualitative feedback from the March to April 2023 cohort (*n* = 4) involved a 5-point Likert scale and comments covering 10 domains. The feedback focused on aspects such as induction, orientation, learning objectives, patient assessment, procedural skills, supervisor feedback, access to resources, timetables, and the overall experience. An average total score was calculated.

Subsequently, strategies were implemented for the April to May 2023 cohort based on the feedback. Weekly check-ins, updated timetables, team introductions, additional teaching sessions, and opportunities for case presentations were among the interventions.

Quantitative and qualitative feedback from the April to May 2023 cohort (*n* = 4) were collected and compared with the previous cohort's feedback.

**Results:**

The feedback scores demonstrated improvement, with the average total score increasing from 4.1/5 (82%) in March – April 2023 to 4.7/5 (94%) in April–May 2023. Students praised the helpful staff, opportunities to present cases, and the tailored and useful nature of the placement. Feedback on improvements included addressing vague timetables, unannounced cancellations of teaching sessions, and limited opportunities for case presentations.

**Conclusion:**

This quality improvement project demonstrated that the targeted interventions helped enhance the educational experience of medical students during their psychiatry placement. The increased feedback scores underscore the positive impact of targeted interventions. The findings emphasize the importance of continuous quality improvement in medical education, ensuring a more positive and enriching experience for medical students in Psychiatry rotations.

